# Potentially inappropriate home medications among older patients with cardiovascular disease admitted to a cardiology service in USA

**DOI:** 10.1186/s12872-017-0623-1

**Published:** 2017-07-17

**Authors:** Marwan Sheikh-Taha, Hani Dimassi

**Affiliations:** 1 Department of Pharmacy Practice, Lebanese American University, Beirut, Lebanon; 2 Department of Pharmaceutical Sciences, Lebanese American University, Beirut, Lebanon

**Keywords:** Beer’s criteria, Cardiovascular disease, Geriatrics, Potentially inappropriate medications

## Abstract

**Background:**

The use of potentially inappropriate medications (PIMs) may pose more risks than benefits to patients and is a major factor contributing to the likelihood of serious adverse drug reactions and negative health outcomes among older patients.

**Methods:**

A retrospective chart review was conducted in a tertiary care center in USA where home medications of the older patients were reviewed and analyzed upon hospital admission over three months, from March till May 2016. Inclusion criteria were age of 65 years and above, history of cardiovascular disease, and admission to the cardiology service. The aim of our study was to determine the frequency and factors associated with PIMs, by applying the updated Beers 2015 criteria.

**Results:**

A total of 404 patients were included in the study and were taking a total of 4669 medications at home, an average of 11.6 ± 4.5 medications per patient. The proportion of PIMS was 20% of all medications reported, with an average of 2.4 PIM per patient, and 87.4% of patients were receiving at least one PIM. Significant association was found between use of PIMs and number of home medications, female gender, and number and types of comorbidities. Comorbidities associated with more PIMs were heart failure, atrial fibrillation/flutter, history of falls/fractures, cerebrovascular accident, and depression. The most commonly prescribed PIMs were: drugs that may exacerbate or cause syndrome of inappropriate antidiuretic hormone secretion or hyponatremia (29.7%), scheduled use of proton pump inhibitors (PPIs) > 8 weeks in non-high-risk patients (11.3%), and benzodiazepines (8.1%).

**Conclusions:**

A high prevalence of PIMs in older patients with cardiovascular disease was observed. Provider education and detailed assessment of medication lists upon hospital admission by multidisciplinary teams can help in preventing the use of PIMs.

## Background

The elderly population is very fragile and prone to medication-related problems as a result of multiple chronic diseases and conditions, poly-pharmacy, and age-related changes in the pharmacokinetics and pharmacodynamics of drugs [1–4]. Consequently, the selection of appropriate medications in this population may be challenging and a complex process, leading to increased risk of inappropriate prescribing. In addition, the elderly account for a substantial portion of all health care resource use and medication costs which underscore the importance of recognizing and preventing drug-related problems in this population [5].

Potentially inappropriate medication (PIM) is defined as “a drug in which the risk of an adverse event outweighs its clinical benefit, particularly when there is a safer or more effective alternate therapy for the same condition” [6]. Several tools have been developed to identify PIMs in the elderly, the most known and frequently used being Beer’s criteria, developed by Mark Beers, MD, in the United States in 1991. The American Geriatrics Society has updated the “Beers Criteria for Potentially Inappropriate Medication Use in Older Adults” in 2015, which includes an explicit list of PIMs best avoided in older adults and in those with certain diseases or syndromes, drugs that should be prescribed at reduced dosage or with caution or carefully monitored, and select drug–drug interactions documented to be associated with harms in older adults [7]. The Beers 2015 criteria were developed by a panel with recognized expertise in geriatric medicine, nursing, pharmacy practice, research, and quality measures.

Numerous studies have examined the prevalence of PIMs in the elderly worldwide; in the United States and Canada, the prevalence ranged between 14% to 37%, while in Europe it ranged between 23% and 43% [8].

The older population has multiple manifestations of cardiovascular disease and most of the medications prescribed to treat their cardiovascular diseases are taken for a long period of time due to the chronic nature of their diseases such as hypertension, coronary artery disease, heart failure, and atrial fibrillation/flutter. A careful search of the available literature revealed few studies on PIMs in patients with cardiovascular diseases. The objective of our study was to determine the frequency and factors associated with potentially inappropriate home medications in older patients with cardiovascular diseases admitted to the cardiology service in a tertiary care teaching center in USA, by applying the updated Beers 2015 criteria.

## Methods

A retrospective chart review was conducted in a tertiary care teaching center, Huntsville Hospital, Alabama, USA, over a three month period, between March and May 2016. Home medications of the older patients were reviewed and analyzed upon hospital admission. Inclusion criteria were age of 65 years and above, history of cardiovascular disease, and admission to the cardiology service. Data collected included, demographic data, condition or disease causing hospital admission, comorbidities, serum creatinine values, height, and weight. Estimation of glomerular filtration rate was calculated using the Cockroft-Gault formula. The updated Beers 2015 criteria were used to identify the PIMs among the study participants. The study was approved by the Institutional Review Board.

Data collected from medical records were recorded on a spreadsheet and analyzed using SPSS version v23 (BM SPSS Statistics for Windows, Version 23.0. Armonk, NY: IBM Corp). Descriptive statistics was expressed as mean and standard deviations for numerical data, and frequencies and percentages for categorical data. The total number of home medication was calculated by summing the number of medication reported by each participant, and the proportion of PIMs was determined by dividing the total number of PIMs by the computed total. The prevalence of PIM was calculated at the patient level, where the number of patients with at least one PIM was considered the numerator and the total number of patients as the denominator. Numerical variables were assessed for normal distribution by inspecting their histograms, boxplots, Q-Q plots, as well as comparing means to medians. A multivariate linear regression was used to estimate the association of multiple factors/determinants with the number of PIMs per patient. The linear coefficient, their corresponding standard errors and *p*-values were reported. Statistical significance was declared at *p* < 0.05.

## Results

A total of 404 patients were admitted to the cardiology service and met inclusion criteria during the study period, of which 211 (52.2%) were males and 193 (47.8%) were females. The mean age was 76.6 ± 7.4 years and at the time of hospital admission, patients had a mean of 6.1 ± 2.4 comorbidities. Table 1 describes patient characteristics and Table 2 describes their comorbidities. Several comorbidities were documented in patients, of which hypertension was the most common (75.0%), followed by dyslipidemia (52.2%), coronary artery disease (49.5%), and heart failure (41.8%). Patients were taking a total of 4669 medications at home; an average of 11.6 ± 4.5 medications per patient. The proportion of PIMs among home medications was 20%, with an average of 2.4 PIM per patient, with 87.4% of patients receiving at least one PIM. Table 3 describes number of home medications and PIMs.

**Table 1:** Patient characteristics

General characteristics	Number	Percent
Age (years)		
65–69	83	20.5
70–74	89	22.0
75–79	86	21.3
80–84	82	20.3
85–89	40	9.9
90+	24	5.9
Mean (SD)	76.6 ± 7.4	
Total	404	
Gender		
Male	211	52.2
Female	193	47.8
Reason for admission		
Rule out cardiac etiology	31.4	31.4
Decompensated heart failure	19.8	19.8
Acute coronary syndrome	16.9	16.9
Atrial fibrillation/flutter	10.6	10.6
Syncope	4.0	4.0
Bradycardia	3.2	3.2
Length of stay		
1–2 days	93	23.5
3–4 days	129	32.6
5–6 days	69	17.4
≥ 7 days	105	26.5
Mean, Median	5.3, 4.0

**Table 2:** Number of types of comorbidities in the same

Comorbidities characteristics	Number	Percent
Number of comorbidities		
1–3	45	11.1
4	74	18.3
5	64	15.8
6	54	13.4
7	61	15.1
8	45	11.1
9+	61	15.1
Mean (SD)	6.1 (2.4)	
Type of comorbidity		
Hypertension	303	75.0
Dyslipidemia	211	52.2
Coronary artery disease	200	49.5
Heart failure	169	41.8
Atrial fibrillation/flutter	145	35.9
Diabetes mellitus	143	35.4
Chronic renal disease	115	28.5
COPD	84	20.8
Gastro-esophageal reflux disease	79	19.6
Hypothyroidism	78	19.3
Cancer	77	19.1
Benign prostatic hyperplasia	65	16.1
Arthritis	63	15.6
Cardiac valve disease	61	15.1
Cerebrovascular accident	52	12.9
Anemia	47	11.6
Peripheral vascular disease	47	11.6
Sleep apnea	39	9.7
Depression	37	9.2
Gout	35	8.7
Pulmonary hypertension	30	7.4
Anxiety	29	7.2
Asthma	26	6.4
Obesity	26	6.4
Dementia	25	6.2
GI bleed/ulcer	25	6.2
Deep venous thrombosis / pulmonary embolism	25	6.2
Neuropathy	24	5.9

**Table 3:** Number of home medication and PIMs

	Number	Percent
Number of medications		
0	2	0.5
1–5	33	8.2
6–7	42	10.4
8–9	49	12.1
10–11	79	19.6
12–13	81	20.0
14–15	47	11.6
16 +	71	17.6
Mean (SD)	11.6 (4.5)	
Total number of home medications	4669	
Number of PIMs		
0	51	12.6
1	92	22.8
2	98	24.3
3	66	16.3
4	49	12.1
5	29	7.2
6	12	3.0
7	3	0.7
8	3	0.7
9	1	0.2
Mean (SD)	2.4 (1.7)	
Total number of PIMs	953	
Proportion of PIM over home medication: Mean (SD)	20% (19.4)	

The most commonly prescribed PIMs were: drugs that may exacerbate or cause syndrome of inappropriate antidiuretic hormone secretion (SIADH) or hyponatremia (29.7%), followed by scheduled use of proton pump inhibitors (PPIs) > 8 weeks in non high-risk patients (11.3%), benzodiazepines (8.1%), and cardiovascular agents that should be avoided or dose reduced due to varying levels of kidney function (6.9%) (Table 4).

**Table 4:** Most common PIMs among home medications

Common PIMs	Number	Percent
Drugs that may exacerbate or cause SIADH or hyponatremia *(including diuretics, SSRIs, SNRIs, and antipsychotics)*	283	29.7
Scheduled use of PPIs >8 weeks in non-high risk patients	108	11.3
Benzodiazepines	77	8.1
Cardiovascular agents that should be avoided or dose reduced due to varying levels of kidney function (including NOACs and potassium sparing diuretics)	66	6.9
CNS and analgesic agents that should be avoided or dose reduced due to varying levels of kidney function (including duloxetine, gabapentin, pregabalin, and tramadol)	53	5.6
Combination of opioid analgesics with ≥2 other CNS active agents	39	4.1
Anticholinergics	38	4.0
H2 antagonists that should be dose reduced due to varying levels of kidney function	29	3.0
Digoxin used as first line therapy for atrial fibrillation / flutter or heart failure or dose >0.125 mg/d	23	2.4
Chronic use of non-cyclooxygenase-selective NSAIDS	23	2.4
Skeletal muscle relaxants	23	2.4

*CNS* central nervous system, *GI* gastrointestinal, *NSAIDs* non-steroidal anti-inflammatory drugs, *NOACs* novel oral anticoagulants, *PPI* proton pump inhibitors, *SIADH* syndrome of inappropriate antidiuretic hormone secretion, *SNRIs* serotonin and norepinephrine reuptake inhibitors, *SSRIs* selective serotonin re-uptake inhibitors

Significant association was found between the number of PIMs per patient and number of home medications, female gender, and number of comorbidities. Where females were observed to have on average 0.4 more PIMs than the males (beta = 0.399 SE = 0.135, *p*-value = 0.003). The number of PIMs increases by a factor of almost 0.2 (beta = 0.178, *p*-value < 0.001) for every additional home medication. Furthermore, comorbidities associated with more PIMs were heart failure, atrial fibrillation/flutter, history of falls or fractures, cerebrovascular accident, and depression. The largest increase in the number of PIMs was for those with history of falls/fractures where these patients were found to have on average more than 1 additional PIM as compared to the rest of the sample (beta = 1. 275, *p* = 0.014) and depression with similar increase (beta = 1.1 and *p*-value <0.001). The model explained 40% of the variation in the number of PIM reported (Adj R^2^ = 04) (Table 5).

**Table 5:** Multivariate regression of factors associated with PIMs

Factors	B	Std. Error	*p*-value
Gender (Female)	0.399	0.135	0.003
Number of home medications	0.178	0.015	<0.001
Heart failure	0.273	0.142	0.056
Atrial fibrillation/flutter	0.468	0.142	0.001
History of falls/fractures	1.275	0.517	0.014
Cerebrovascular accident	0.565	0.200	0.005
Thromboembolism	0.559	0.287	0.052
Depression	1.104	0.236	<0.001
Adjusted R^2^ = 40%			

B: regression coefficient

R^2^: the coefficient of determination

## Discussion

To our knowledge, this is the first study to evaluate the use of potentially inappropriate home medications among patients with cardiovascular disease admitted to a cardiology service. Inappropriate use of medications is more likely to occur in older patients since they are more likely to receive several medications concomitantly due to chronic diseases, and/or multiple health problems. In addition, age-related changes in the pharmacokinetics and pharmacodynamics of drugs can play a role in inappropriate prescribing. In our study, patients were taking an average of 11.6 medications at home and had a mean of 6.1 comorbidities, at least one of which was a cardiovascular disease. Our study suggests a very high level of exposure to PIMs among older patients with cardiovascular diseases according to the updated 2015 Beers criteria; the prevalence of PIMs among home medications was 20%, and 87.4% of patients had at least one PIM, with an average of 2.4 PIMs per patient. Beers criteria PIMs have been found to be associated with poor health outcomes, including confusion, falls, and mortality [9, 10].

It would be very challenging to compare the results of our study that involved patients with cardiovascular diseases to other available studies knowing that the prevalence of PIMs varies substantially depending on the measures and definitions used, as well as the characteristics of the studied group of patients, including clinical settings and type of comorbidities. Another factor causing a variation in the prevalence of PIMs among studies could be the different prescribing habits in different countries. Nevertheless, the results of our study are consistent with those conducted in USA and Canada [8].

The most commonly prescribed PIMs in our study were drugs that may exacerbate or cause SIADH or hyponatremia (29.7%). According to Beers criteria, the use of this category of medications is to be used with caution in older adults (not to be avoided) and clinicians are strongly advised to monitor sodium level closely when starting or changing dosages of these medications. The most commonly used medications that belong to this category of PIMs in our study were the diuretics (a total of 175 PIMs), followed by selective serotonin re-uptake inhibitors (SSRIs), and serotonin and norepinephrine reuptake inhibitors (SNRIs). The high use of diuretics is expected in our patients as 41.8% suffered from heart failure, a condition that is commonly associated with fluid overload [11]. Furthermore, 16.4 of our patients suffered from depression or anxiety which is an indication for using SSRIs or SNRIs.

The unnecessary use of PPIs accounted for 11.3% of the PIMs in our study. PPIs continue to be among the most commonly used group of drugs in many countries and many patients take them chronically without an indicated diagnosis. It is important that prescribers carefully consider the use of PPIs in older adults and monitor their continued use to prevent the unnecessary cost, drug-drug interactions and side effects, including risk of *Clostridium difficile* infection, bone loss, and fractures [12].

Benzodiazepines were also among the most commonly prescribed PIMs. With aging, more elderly suffer from insomnia, anxiety, and other psychiatric disorders leading physicians to prescribe benzodiazepines. The 2015 Beers criteria recommends the avoidance of this class of medications in older adults due to the increased risk of cognitive impairment, falls, fractures, delirium, and motor vehicle crashes. Few older patients and prescribers seem to be willing to try non-pharmacologic therapy or other medications instead of benzodiazepines.

Age-associated loss of kidney function is a well-known fact and many cardiovascular medications including potassium sparing diuretics and anticoagulants, specifically novel oral anti-coagulants (NOACs), need dose adjustment or should be avoided in patients with reduced kidney function [13]. In our study 6.9% of the PIMs were mostly attributed to NOACs followed by potassium sparing diuretics (mostly spironolactone) in patients with low creatinine clearance at which action was required.

There was a significant association between use of PIMs and number of home medications, number of comorbidities, and female gender. Increase in the number of comorbidities, which is expected with aging, necessitates prescribing more drugs to patients which can lead to more PIMs. Poly-pharmacy has been shown in several studies to be associated with PIMs [14–16]. Furthermore, our findings that females have 0.4 PIM more than males is consistent with other studies, a fact that deserves further investigation [17–21]. While neither socioeconomic status nor comorbidity explained sex differences in one study, biological and social factors were suggested to play a role by some investigators [19, 21].

In our study, cardiovascular diseases associated with more PIMs were heart failure, atrial fibrillation/flutter, and cerebrovascular accident. This should alert cardiologists to carefully prescribe and monitor medications commonly given to treat those conditions.

Strategies that were shown to be effective in reducing the use of PIMs include provider education and detailed assessment of home medication lists upon hospital admission by a multidisciplinary team, which involves geriatricians and specialized clinical pharmacists [22–25]. Poly-pharmacy should provoke attempts to stop unnecessary medications and drugs that are prone to be prescribed inappropriately; CNS-active drugs, PPIs, NSAIDs, NOACs, diuretics, and digoxin should be carefully evaluated and monitored. This should be followed by proper medication reconciliation upon hospital discharge.

Our study has several limitations. First, it was a retrospective study. Second, there was no individualized evaluation of risk and benefit for every single patient. The PIMs could have been prescribed due to poor tolerance of alternative medications or possibly to avoid certain drug-drug interactions. Third, our study was limited to one hospital setting despite being ranked and recognized among the best hospitals in America.

## Conclusion

A high prevalence of PIMs in older patients with cardiovascular disease was observed. Provider education and detailed assessment of medication lists upon hospital admission by multidisciplinary teams can help in preventing the use of PIMs and consequently, decrease the potential for drug-drug interactions, risk of serious adverse drug reactions, and hospitalization.

## Abbreviations

NOACs: Novel oral anti-coagulants
PIM: Potentially inappropriate medication
PPIs: Proton pump inhibitors
SIADH: Syndrome of inappropriate antidiuretic hormone secretion
SNRIs: Serotonin and norepinephrine reuptake inhibitors
SSRIs: Selective serotonin re-uptake inhibitors
